# Spinon confinement and a sharp longitudinal mode in Yb_2_Pt_2_Pb in magnetic fields

**DOI:** 10.1038/s41467-019-08715-y

**Published:** 2019-03-08

**Authors:** W. J. Gannon, I. A. Zaliznyak, L. S. Wu, A. E. Feiguin, A. M. Tsvelik, F. Demmel, Y. Qiu, J. R. D. Copley, M. S. Kim, M. C. Aronson

**Affiliations:** 1 Department of Physics and Astronomy, Texas A&M University, College Station, TX 77843 USA; 2 Condensed Matter Physics and Materials Science Division, Brookhaven National Laboratory, Upton, NY 11973 USA; 3 Quantum Condensed Matter Division, Oak Ridge National Laboratory, Oak Ridge, TN 37830 USA; 4 Department of Physics, Northeastern University, Boston, MA 02115 USA; 5 ISIS Facility, Rutherford Appleton Laboratory, Didcot, OX11 0QZ UK; 6 NIST Center for Neutron Research, National Institute of Standards and Technology, Gaithersburg, MD 20899 USA; 7 Department of Physics and Astronomy, Stony Brook University, Stony Brook, NY 11794 USA; 8 Present Address: Stewart Blusson Quantum Matter Institute, University of British Columbia, Vancouver, BC V6T 1Z4 Canada; 9 Present Address: Department of Physics, South University of Science and Technology of China, 518055 Shenzhen, China

## Abstract

The fundamental excitations in an antiferromagnetic chain of spins-1/2 are spinons, de-confined fractional quasiparticles that when combined in pairs, form a triplet excitation continuum. In an Ising-like spin chain the continuum is gapped and the ground state is Néel ordered. Here, we report high resolution neutron scattering experiments, which reveal how a magnetic field closes this gap and drives the spin chains in Yb_2_Pt_2_Pb to a critical, disordered Luttinger-liquid state. In Yb_2_Pt_2_Pb the effective spins-1/2 describe the dynamics of large, Ising-like Yb magnetic moments, ensuring that the measured excitations are exclusively longitudinal, which we find to be well described by time-dependent density matrix renormalization group calculations. The inter-chain coupling leads to the confinement of spinons, a condensed matter analog of quark confinement in quantum chromodynamics. Insensitive to transverse fluctuations, our measurements show how a gapless, dispersive longitudinal mode arises from confinement and evolves with magnetic order.

## Abstract (web summary)

The behavior of quantum magnets depends strongly on the effective dimensionality of the inter-spin interactions. Here the authors tune the influence of two- and three-dimensional couplings in Yb_2_Pt_2_Pb with a magnetic field and hence control the behavior of emergent one-dimensional excitations.

## Introduction

The one-dimensional (1D) *XXZ* Hamiltonian for quantum spin chains given by Eq. (1) is a cradle of exactly solvable quantum theory models of interacting many-body systems^1^. The exact solution features purely quantum-mechanical entities and concepts such as fractional excitations and the quantum-critical Luttinger-liquid state^2–9^. The Hamiltonian considers the components $S_i^\alpha$ (*α* = *x*, *y*, *z*) of a spin angular momentum operator, **S**_*i*_ (*S* = 1/2) at site *i* on a 1D chain, with ${\cal J}$ a nearest neighbor exchange coupling for *x*, *y* spin components, Δ a uniaxial coupling anisotropy, and **H** magnetic field (with *g* and *μ*_B_ the Lande g-factor and Bohr magneton respectively),

1 $${\cal H} = {\cal J}\mathop {\sum}\limits_i \left( {S_i^xS_{i + 1}^x + S_i^yS_{i + 1}^y} \right) + {\mathrm{\Delta }}S_i^zS_{i + 1}^z - g\mu _{\mathrm{B}}\mathop {\sum}\limits_i {\kern 1pt} {\bf{H}} \cdot {\bf{S}}_i.$$

**Fig. 1:**
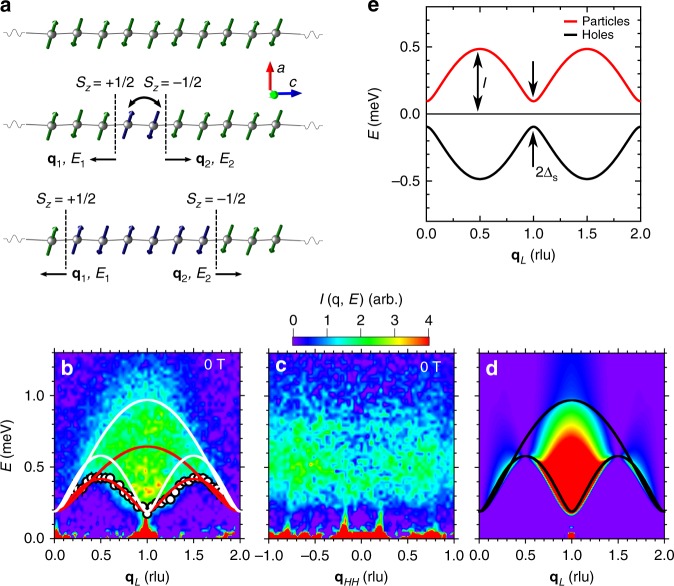
Spinons on decoupled chains. **a** An anisotropic AFM spin chain (top). If two spins are interchanged, two domain walls are created between the original antiferromagnetic domain (green) and a new domain (blue) (middle). Those domain walls form the basis for the propagating states carrying energy and momentum quanta (bottom). **b** The magnetic excitation spectrum and dispersion along the **q**_*L*_ direction in reciprocal space measured at *T* = 0.1 K, summed over −1 ≤ **q**_*HH*_ ≤ 1 rlu. The lower boundary of the spectrum (white circles) is shown along with the boundaries of the two spinon continuum obtained by fitting the lower boundary (red lines, Δ = 3.46) and comparing the total measured spectrum to theory (white lines, Δ = 2.6)^26^. The color scale for parts **b**–**d** is shown above part **c**. Error bars represent one standard deviation. **c** The magnetic excitation spectrum along the **q**_*HH*_ direction in reciprocal space measured at *T* = 0.1 K, summed over 0 ≤ **q**_*L*_ ≤ 2 r.l.u. **d** The spinon spectrum obtained from tDMRG calculations for the *XXZ* model Eq. (1) with Δ = 2.6 on the 96-site chain. The continuum boundary (black lines) is the same as that shown in **b** for Δ = 2.6. **e** The dispersions of particle-like (red) and hole-like (black) spinons, symmetric about *E* = 0 in zero magnetic field, are sketched with the real Δ = 2.6 parameters for Yb_2_Pt_2_Pb. The bandwidth parameter *I* and the spinon gap Δ_S_ are indicated by arrows, with 2Δ_S_ the energy separation between the particle and hole bands at **q**_*L*_ = 0, 1, and 2 rlu

The low energy excitations of this model Eq. (1) are spin-1/2 quasiparticles called spinons. In the limit of strong Ising anisotropy, ${\mathrm{\Delta }} \gg 1$, spinons can be visualized as domain walls in an antiferromagnetically ordered ground state of the chain (Fig. 1a). Angular momentum conservation mandates that spinons are always created in pairs, such that each spinon carries a fraction, ±1/2, of the angular momentum change, Δ*S*^*z*^ = 0, ±1, required to initially introduce the domain walls in an infinite chain. Since moving these domain walls is an energy and angular momentum conserving process, the walls will propagate freely, carrying the quanta of energy, *E*, and linear momentum, *q*, introduced by their creation (Fig. 1a). The physics contained in Eq. (1) leads directly to the separation of the spin from other electronic degrees of freedom, mapping directly onto that of the Luttinger liquid for −1 ≤ Δ ≤ 1^2,6–10^.

Coupling the chains described by Eq. (1) leads to new and emergent physics. Analogous to quark confinement in quantum chromodynamics^4,5,8^, the dimensional crossover from 1D chains to 3D coupled chains leads to quasiparticle confinement, thereby stabilizing long range magnetic order at temperature *T* > 0. A new excitation of the longitudinal degree of freedom of the order parameter is predicted when the interchain coupling is weak^6^. These phenomena have been the subject of a considerable amount amount of recent experimental work in *XXZ* spin chain materials^11–15^. Like a similar longitudinal mode previously observed near the critical point in a system of coupled spin-1/2 dimers^16,17^, this excitation can be interpreted as a condensed matter analog of the Higgs boson^18^.

Here, we report neutron scattering experiments on the one dimensional rare-earth antiferromagnet Yb_2_Pt_2_Pb to investigate these fundamental processes in detail, using an external magnetic field as a tuning parameter.

## Results

### Inelastic neutron scattering on Yb_2_Pt_2_Pb

Yb_2_Pt_2_Pb is a metal with a planar crystal structure where orthogonal pairs of Yb ions form a Shastry-Sutherland lattice (SSL) motif in the tetragonal **a**−**b** plane^19–25^. High resolution neutron scattering experiments recently showed that the physics of 4*f*-orbital overlaps leads to unusual consequences for the magnetism in Yb_2_Pt_2_Pb^26^. The low energy magnetic excitations are spinons, having a quantum continuum for momentum along the chain direction **q**_*L*_ that can be measured with inelastic neutron scattering^27^, with an excitation bandwidth that is considerably larger than the excitation gap (Fig. 1b). For momenta in the interchain **q**_*HH*_ direction (Fig. 1c), the continuum is entirely flat, indicating that the spinons are completely incoherent between the chains.

In zero field, our measurements agree well with time-dependent density matrix renormalization group (tDMRG) calculations^28^ for the *XXZ* model Eq. (1) (Fig. 1d), although experiment indicates that the spectral weight is spread throughout the spinon Brillouin zone (BZ) more evenly and to higher energies than these calculations predict, suggesting non-negligible next-neighbor coupling^26^. Comparisons of our data to theory indicate only a modest anisotropy, Δ ~ 2–3. It is clear that the *XXZ* Hamiltonian Eq. (1) is an appropriate description for Yb_2_Pt_2_Pb despite the large and orbitally dominated moment of the Yb ions. Due to their Kramers doublet ground state of almost pure $| {J,m_J} \rangle = | {7{\mathrm{/}}2, \pm 7{\mathrm{/}}2} \rangle$, the Yb moments have a pseudospin **S** = 1/2 character^26,29^. Rather than quenching the quantum spin dynamics, the strong Ising magnetic anisotropy imposed by the crystal electric field acting on the *f*–orbital wave function instead singles out the longitudinal excitation channel in two orthogonal sublattices of 1D chains with moments oriented along the (110) and (${\bar{\mathrm 1}}$10) crystal directions.

The essential features of the quantum continuum can be understood by noting that each spinon carries spin 1/2, and so the angular momentum selection rules dictate that neutron scattering in Yb_2_Pt_2_Pb measures two-spinon states where the total spin is zero, with one spinon in each spin state, ±1/2. In order to describe the boundaries of the two-spinon continuum, it is convenient to adopt the language of particles and holes occupying the fermionic spinon dispersion along the chain direction, $E_{{\mathrm{p,h}}} = \pm \left( {I^2\,{\mathrm{sin}}^2\left( {\pi {\bf{q}}_L} \right) + {\mathrm{\Delta }}_{\mathrm{S}}^2{\mathrm{cos}}^2\left( {\pi {\bf{q}}_L} \right)} \right)^{1/2}$, 0 ≤ **q**_*L*_ < 1 rlu, where Δ_S_ is an energy gap brought on by the *XXZ* anisotropy Δ > 1, and *I* defines the dispersion bandwidth and encodes the coupling ${\cal J}$ (Fig. 1e)^1,26,30,31^. In place of electric charge, these particles and holes each carry a half unit of spin angular momentum. The boundaries of the two-spinon continuum are defined by the extremal energy and momentum conserving combinations of one particle and one hole, and they are shown in Fig. 1b for both Δ = 2.6 and 3.46, the range of values determined in previous work^26^ (see Supplementary Note 1). At zero magnetic field, the chemical potential is in the middle of the gap separating the particle and the hole bands, which describes the antiferromagnetic (AFM) state with zero total spin, *S*^*z*^ = 0. The size of the *T* = 0 ordered moment implied by the *XXZ* anisotropy is consistent with our measurements at *T* = 0.1 K, within the precision of our data^26^. This implies that the interchain coupling responsible for moving the Néel temperature away from *T* = 0, the value predicted by the *XXZ* model, to *T*_N_ = 2.07 K is less than both the intrachain exchange ${\cal J} = 0.206\,{\mathrm{meV}}$ and the spinon gap Δ_S_ = 0.095 meV, the dominant 1D energy scales. The flat dispersion of the excitations between the chains in zero field (Fig. 1c), despite the apparent ladder geometry of the crystal structure, suggests that the effect of interchain interactions on low energy excitations is quenched when Δ_S_ is nonzero.

**Fig. 2:**
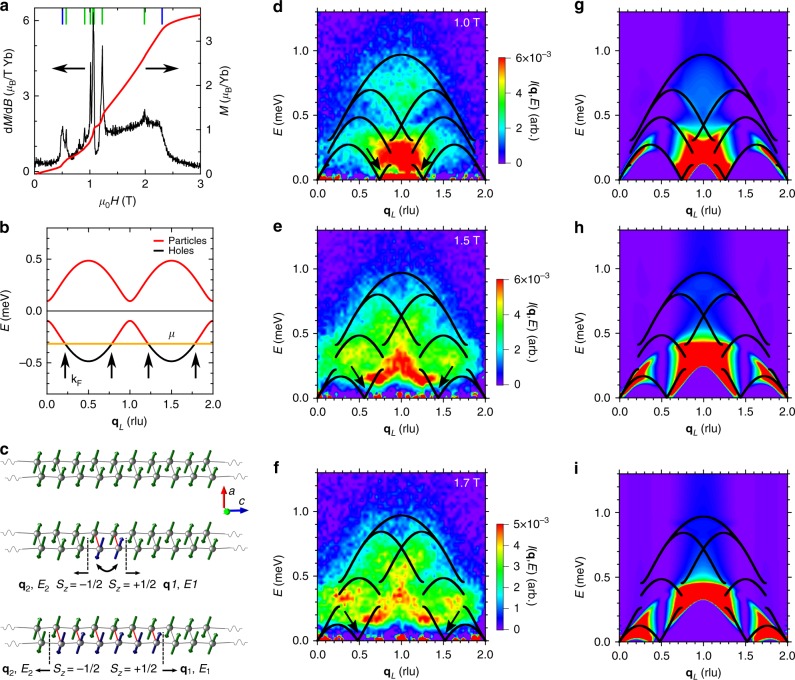
Spinons in a magnetic field. **a** The magnetic field dependence of the static magnetization at *k*_B_*T* < Δ_S_ [red, right axis, **H**||(110)] shows several discontinuous jumps (blue and green dashes), corresponding to transitions among different 3D ordered phases that are more clear in d*M*/d*μ*_0_*H* (black, left axis)^23,25^. These phases correspond to different ways that magnetic moments arrange into registry minimizing the energy of magnetic dipole interactions between the Yb moments. **b** When a magnetic field is applied along the chain direction, the chemical potential *μ* = −*gμ*_B_*HS*^*z*^ (yellow) is lowered, emptying part of the hole band when |*μ*| > |Δ_S_|. *μ* crosses the hole dispersion at four points in the Brillouin zone (black arrows), defining the Fermi wavevector **k**_F_. **c** Two AFM ordered, 1D spin chains (top). If two spins on one chain are interchanged, two domain walls are formed between the original domain (green) and a new one (blue). The new domain frustrates the interchain interaction, represented by the red interchain bonds. This frustration creates a linear potential confining low energy spinons to bound states (bottom). **d**–**f** The magnetic excitation spectrum and its dispersion along the **q**_*L*_ direction in reciprocal space measured at *T* = 0.1 K and *μ*_0_*H* = 1.0 T (**d**), 1.5 T (**e**), and 1.7 T (**f**), summed over −1 ≤ **q**_*HH*_ ≤ 1 rlu. The dispersions for the extremal combinations of particles and holes are shown (black lines) (See Supplementary Note 2). The spinon bound states are manifest from the enhanced low energy spectral weight around 1 ± 2**k**_F_ (black arrows). **g**–**i** The spinon spectrum computed using tDMRG calculations for the *XXZ* model Eq. (1) on a 96-site chain with Δ = 2.6 at equivalent chain magnetizations as (**d**–**f**), shown on the same color scale. The dispersions for the extremal combinations of particles and holes are also shown as black lines

A magnetic field along the **z** (110) direction introduces the Zeeman term $- g\mu _{\mathrm{B}}H\mathop {\sum}\nolimits_i {\kern 1pt} S_i^z$ to Eq. (1), which lowers the chemical potential, *μ* = −*gμ*_B_*HS*^*z*^. The potential needed to close the energy gap for creating a hole on the spinon dispersion is |*μ*| = Δ_S_ = 0.095 meV. Taking *g* = 7.3^26^, |*μ*| = Δ_S_ corresponds to a critical field of *μ*_0_*H* = 0.5 T. An abrupt increase in the bulk magnetization is seen at this field when oriented parallel to the magnetic moments of either sublattice at temperatures *k*_B_*T* < Δ_S_ (Fig. 2a)^23,25^. On the other extreme of the magnetization, when |*μ*| > *I*, the entire hole band lies above the chemical potential, the field having transformed all holes to particles. Particle-hole pairs can no longer be produced, quenching spinon excitations, and producing a ferromagnetic (FM) state. The saturation field in Yb_2_Pt_2_Pb is 2.3 T, precisely the field needed for *μ* = 0.485 meV, the value of *I* when Δ = 2.6, the number obtained by requiring that Eq. (1) provides best description of the entire *μ*_0_*H* = 0 excitation spectrum^26^. The comparison is less favorable when Δ is taken to be 3.46, the value derived directly from fitting the lower boundary of the continuum. As the static properties correspond to an integration over all energies, it is not surprising that they are better captured by Δ = 2.6 and we adopt this value of Δ here.

At intermediate fields, 0.5 < *μ*_0_*H* < 2.3 T, the hole band is partially emptied. The chemical potential crosses the hole dispersion at four points in the spinon BZ (Fig. 2b), defining a Fermi wavevector **k**_F_ that directly links particle and hole states. There are now eleven unique extremal states made from a single particle and hole, rather than the three that are possible in zero field. The boundaries of the two spinon continuum change dramatically, as the possible extremal states are heavily influenced by the restrictions of the hole energies and momenta and the additional phase space occupied by particles.

That is precisely what is measured in the neutron scattering spectra of Yb_2_Pt_2_Pb (Fig. 2d–f). At *μ*_0_*H* = 1.0 T, there is strong scattering concentrated at low energies within a range 1 ± 2**k**_F_ around the BZ center, with a weaker continuum at higher energies. As the magnetic field is increased, the low-energy spectral weight spreads throughout the zone as **k**_F_ increases, with higher energy pockets of spectral weight bounded by the extremal two spinon states comprising the continuum. While the measured continua are in broad agreement with the results of tDMRG calculations performed on isolated chains (Fig. 2g–i), there are marked differences at low energies where interchain interactions are important. The measured lower boundaries are gapped at small energies and also slightly distorted relative to theoretical expectations, with the increased spectral weight indicative of a bound state. This is a direct demonstration of spinon confinement induced by interchain coupling, which is not accounted for in the 1D calculations of Fig. 2g–i, and also shows how a magnetic field tunes the coexistence of confined and free spinons in Yb_2_Pt_2_Pb for *μ*_0_*H* > 0.5 T.

The schematic picture of spinons and their propagation presented in Fig. 1a needs to be modified in the presence of the interchain interactions, where the creation of spinons on one chain leads to frustration of the AFM interactions between the chains. As two spinons separate, the energy of this frustration grows with the number of FM aligned neighbors (Fig. 2c). This provides a linear confining potential, just as quarks are confined by the gluon-mediated strong force in QCD, which also increases with quark separation. When spinons are created with energies above the highest energy level existing in the confining potential introduced by the interchain coupling, these high energy quasiparticles propagate freely within the two spinon continuum, demonstrating the same asymptotic freedom as experienced by unbound quarks^32^. A spinon bound state is observed in a neutron scattering experiment as excess spectral weight of resolution-limited energy width, which prominently appears near 1 ± 2**k**_F_, the two soft spots around the BZ center, below the quantum continuum^10^.

Perhaps the most interesting aspect of these free and confined excitations is how their dispersions develop in the **q**_*HH*_ direction, perpendicular to the chains. Spinons are continually created in pairs on all chains, and in the absence of interchain coupling they are free to propagate. Order and frustration in coupled chains naturally lead to the bound states, which develop an interchain dispersion that reflects the underlying AFM order. Spinons are generated in registry on adjacent chains, thus minimizing the interchain frustration.

**Fig. 3:**
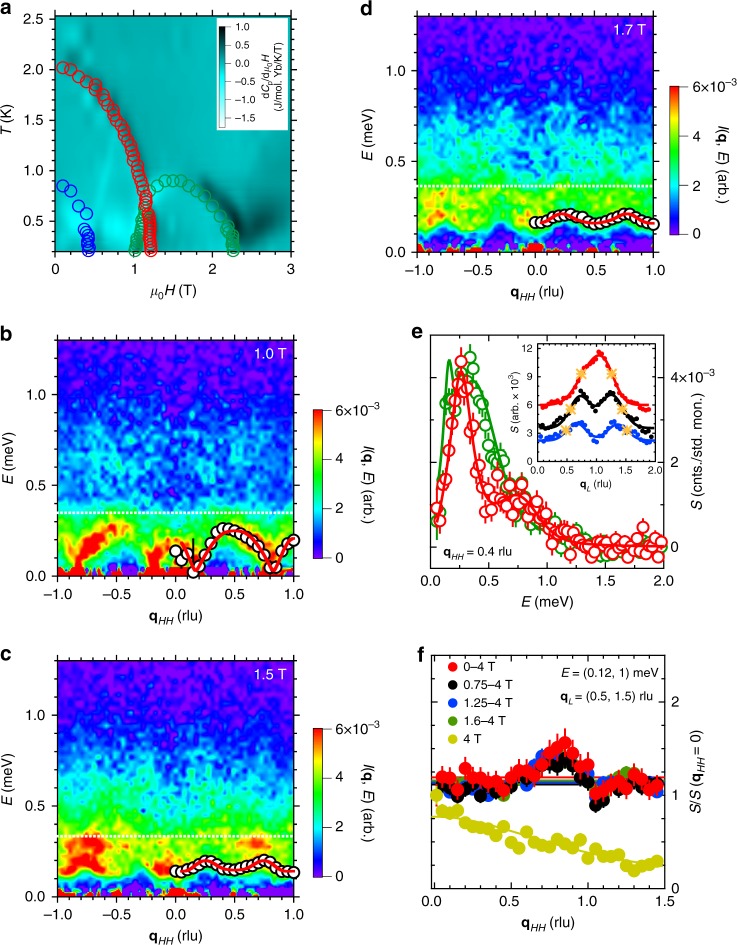
A coherent, longitudinal interchain mode in Yb_2_Pt_2_Pb. **a** The field-temperature phase diagram of Yb_2_Pt_2_Pb deduced from specific heat measurements (see Supplementary Note 3). Symbols representing phase lines of the low field AFM order (red), gapped phase when *k*_B_*T* < Δ_S_ (blue), and second, weaker order (green) are obtained from the magnetic field dependence of the magnetization along the (110) direction at fixed temperatures. Refer to Fig. 2a for an example. Not all of the field-induced AFM phases observed in the magnetization are shown. **b**–**d** The magnetic excitation spectrum along the **q**_*HH*_ direction of reciprocal space measured at *T* = 0.1 K and *μ*_0_*H* = 1.0 T (**b**), 1.5 T (**c**), and 1.7 T (**d**), summed over 0 ≤ **q**_*L*_ ≤ 2 rlu. The fitted dispersion of the interchain mode is shown (white circles), as well as fits to the expression described in the main text (red lines). The mode dispersion and fits are only shown for **q**_*HH*_ > 0 for clarity (see Supplementary Note 4). The continuum boundary appears at 2Δ_M_^33^ (white dashed line). **e** Example cuts of the energy dependence at **q**_*HH*_ = 0.4 rlu measured at *B* = 1.0 (red) and 1.5 T (green). Fits are described in the text. The mode dispersions shown in **b**–**d** are extracted from such fits. (Inset) The integrated intensity of the scattering over the energy of the interchain mode extracted from the fits is shown in parts **b**–**d** as a function of momentum **q**_*L*_ along the chain. Magnetic fields of 1.0 T (red), 1.5 T (black), and 1.7 T (blue) are offset for clarity. **q**_*L*_ = 1±2**k**_F_ is shown at each field (gold stars). **f** The polarization of the excitations follows the projection onto the scattering vector **q** of the magnetic moments contributing to the scattering, indicating that all observed excitations are longitudinal (see Supplementary Note 5). Fields and integration ranges are given in the figure legend; the calculated polarization was averaged within the corresponding **q**_*L*_ integration range. Error bars represent one standard deviation and where not visible are smaller than symbol size

Figure 3a shows a complex phase diagram consisting of several distinct phases that differ markedly in the behavior of the inter-chain dispersion. Most notably, for 0.5 ≲ *μ*_0_*H* ≲ 2.3 T, we observe a new excitation that emerges from the featureless spinon continuum found along **q**_*HH*_ in the gapped zero field Néel phase where the chains are effectively decoupled. This mode resides within the low-energy window of the two spinon bound states, but has a pronounced dispersion in the **q**_*HH*_ (interchain) direction that changes considerably with increasing field (Fig. 3b–d). Remarkably, at 1.0 T the dispersive interchain mode appears nearly gapless, while its intensity is markedly larger than that of the continuum, Figs. 2d and 3b. The mode becomes clearly gapped with increasing fields, while the relative spectral weight of the continuum grows. We model the energy dependence of the scattering at a specific **q**_*HH*_ as a damped harmonic oscillator (DHO) response centered at the mode position and the product of a Lorentzian and step function accounting for the continuum at higher energies, all convolved with the instrument resolution (Fig. 3e). The energy width of the new mode is roughly resolution limited at all fields, and it is always distinguishable from the spinon continuum for fields *μ*_0_*H* ≤ 1.7 T. The connection of the mode and the confined spinon states can be emphasized by integrating over the energies of the mode and plotting the intensity as a function of momentum along the chains (Fig. 3(e-Inset)). At all fields, ≈85% of the spectral weight is concentrated within the momentum range **q**_*L*_ = 1 ± 2**k**_F_.

Importantly, this interchain mode is longitudinally polarized. We confirm its longitudinal character by using the fact that neutron scattering cross-section is uniquely sensitive to magnetic fluctuations that are perpendicular to the wave vector transfer^27^. The intensity measured at 4 T, in the FM state precisely follows the projection of the scattering wave vector on the (110) direction, revealing fluctuations polarized along the in-plane Ising moments, which are insensitive to magnetic fields (Fig. 3f). When this field-independent contribution is subtracted as a background, the resulting field-dependent intensity [Figs. 2 and 3) does not depend on the wave vector orientation in the scattering plane, indicating magnetic fluctuations polarized along the vertical direction, collinear with the magnetic field^26^ (see Supplementary Note 5). At all fields, our measurements unambiguously probe the longitudinal response. The Ising anisotropy of the 7/2, ±7/2 ground state doublet of the Yb moments nearly completely suppresses any transverse magnetic fluctuations from our measurements.

**Fig. 4:**
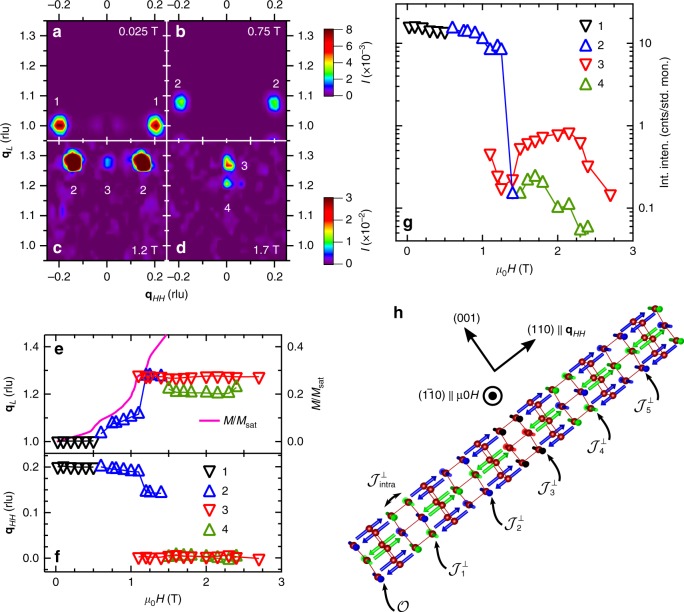
Magnetic order and phase diagram of Yb_2_Pt_2_Pb. **a**–**d**. Time of flight data with energy transfer *E* = 0 in the **q**_*HH*_, **q**_*L*_ plane, measured at *T* = 0.1 K in fields of 0.025 T (**a**), 0.75 T (**b**), 1.2 T (**c**), and 1.7 T (**d**). The magnetic Bragg diffraction peaks in each part of the phase diagram are labeled 1–4. (**e**, **f**) The location in reciprocal space along **q**_*L*_ (**e**) and **q**_*HH*_ (**f**) of the magnetic Bragg scattering as a function of the field in the regions plotted in parts **a**–**d**. Data shown are an average, symmetrized about **q**_*L*_ = 1 and **q**_*HH*_ = 0. The open symbols correspond to the peaks labeled 1-4 in panels **b**–**e**, with black indicating peak 1, blue peak 2, red peak 3, and green peak 4. Magnetization divided by the saturation magnetization *M*/*M*_sat_ as a function of the field along the (110) crystal direction is also shown in panel **e** (pink line, right axis), demonstrating the initial trend, *M*/*M*_sat_ ≈ (**q**_*L*_ − 1) and the concurrence of the jumps in magnetization with the abrupt changes in the position of elastic scattering. (**g**) The intensity of the diffraction peaks in parts **b**–**f**. The AFM order that emerges for *μ*_0_*H* > 1 T is considerably weaker than the low field order, while the low field order falls off very abruptly for *μ*_0_*H* > 1.2 T. Symbols are the same as in panels **e**, **f**. (**h**) The zero field magnetic structure and dipole interactions in Yb_2_Pt_2_Pb. Yb moments in five unit cells are shown along the diagonal, (110) direction, with the interchain couplings, ${\cal J}_n^ \bot$, as indicated. The Yb SSL AFM layers are highlighted (blue and green arrows), with the periodicity given by FM pairs every five unit cells (black and red arrows)

The longitudinal interchain mode changes dramatically over a relatively narrow range of fields as the underlying antiferromagnetic order is weakened and ultimately destroyed. The low temperature **H**−*T* magnetic phase diagram of Yb_2_Pt_2_Pb (Fig. 3a) has several different AFM ordered phases^22–25^. In zero field, there is a five by five periodicity to the order in the tetragonal **a**−**b** plane, evidenced by neutron diffraction peaks that index as **q**_*HH*_ = 0.2 rlu (Fig. 4a)^29^. When the gap Δ_S_ closes at *μ*_0_*H* = 0.5 T, those peaks move from **q**_*L*_ = 1 rlu to incommensurate positions along **q**_*L*_ (Fig. 4b), consistent with the longitudinal component of the spin-spin correlation function probed by our neutron scattering measurements being locked to twice the Fermi wave vector^10^. The ordering wave vector follows 2**k**_F_ in turn, connecting to the softest parts of the excitation spectrum. There are several small and abrupt shifts in the ordering wave vector along both **q**_*L*_ and **q**_*HH*_ (Fig. 4a–f) that coincide with abrupt jumps in the derivative of the low temperature magnetization [Figs. 2a and 4e, f), which manifest changes in 3D magnetic ordering as the magnetic moments re-arrange to minimize the energy of magnetic dipole interactions. For fields *μ*_0_*H* > 1.0 T, there is an emergence of a second incommensurate AFM ordered phase that is accompanied by the swift collapse of the original five by five order for *μ*_0_*H* > 1.2 T and even a third incommensurate order that persists up to the saturation field (Fig. 4c–g). As the new longitudinal interchain mode is an excitation of the underlying order, it is not surprising that it changes so dramatically between 1.0 and 1.5 T.

For 0.5 ≲ *μ*_0_*H* ≲ 1 T, the **q**_*L*_ component of the magnetic Bragg peak follows the magnetization (Fig. 4e), which reflects the position of the spinon Fermi wavevector, 2**k**_F_ (Fig. 2b) (See Supplementary Note 6). Theoretical description of the excitation spectrum in this spin-density-wave (SDW) phase can be obtained by applying bosonization methods for quasi-1D spin-1/2 antiferromagnets^33,34^. The low energy sector of such an antiferromagnet is described by the theory of noninteracting bosonic field *ϕ* governed by the Lagrangian,

2 $$L = \frac{1}{{4\pi }}{\int} {\kern 1pt} dx\left[ {v^{ - 1}\left( {\partial _\tau \phi } \right)^2 + v\left( {\partial _x\phi } \right)^2} \right],$$

where *v* ~ *J*/*a*. The expression for the *z*-component of the spins is

3 $$S^z - M = \frac{1}{{2\pi }}\partial _x\phi + A\,{\mathrm{sin}}[\phi + \pi (1 - 2M)x],\,M = \left\langle {S^z} \right\rangle ,$$

where *A* is an amplitude with dimension of inverse length. A quantitative description of the interchain dispersion requires knowledge of the relevant couplings for the underlying order. In Yb_2_Pt_2_Pb, the interchain interaction is predominantly of dipole-dipole origin. The symmetry of the Yb lattice sites suppresses magnetic dipole interaction between the two orthogonal sublattices, which cancels on the mean field level. On the other hand, due to the Ising nature of Yb magnetic moments the intra-sublattice interactions ${\cal J}_{{\mathrm{intra}}}^ \bot$ involve only *z*- components of effective spins-1/2 (Fig. 4h). Hence, the interchain coupling can be written as, ${\cal J}_m^ \bot {\int} dxS^z(n,x)S^z(m + n,x)$, where *n*,*n* + *m* label different chains. Using (3) and neglecting the marginal terms with derivatives of *ϕ* we get the following contribution to (2),

4 $$L\prime = \mathop {\sum}\limits_{n,m} {\kern 1pt} A^2{\cal J}_m^ \bot {\int} {\kern 1pt} dx\,{\mathrm{cos}}[\phi (n,x) - \phi (m + n,x)].$$

Since fluctuations in 3D do not lead to divergencies, we can expand cosines in *L*′ around its minimum and get the Lagrangian quadratic in *ϕ*. The zero field magnetic structure in Yb_2_Pt_2_Pb suggests couplings up to fifth neighbors in the basal plane. We thus obtain a gapless longitudinal “phason” mode with the interchain dispersion,

5 $$E_{{\mathrm{mode}}}\left( {{\bf{q}}_{HH}} \right)^2 = 2\mathop {\sum}\limits_{n = 1}^5 {\kern 1pt} {\cal J}_n^ \bot \left[ {1 - {\mathrm{cos}}\left( {2\pi n{\bf{q}}_{HH}} \right)} \right],$$

which accurately describes the 1.0 T data in Fig. 3b.

The situation is different at 1 T < *μ*_0_*H* < 2.2 T (region marked by green circles on the phase diagram, Fig. 3a). In this region, the static susceptibility is nonzero and magnetization smoothly increases, but the longitudinal interchain mode is gapped and magnetic Bragg peaks are locked to *q*_*L*_ ≈ 0.29 rlu and do not change their *q*_*L*_ positions with field (Fig. 4e). In principle, this presents a puzzle, which could be resolved by assuming that the transverse components of the effective spins order in a spiral configuration, while the *z*-component has a field independent ordering wave vector. Since neutrons do not register transverse fluctuations in Yb_2_Pt_2_Pb, the corresponding Goldstone mode is invisible. In order to describe the gapped longitudinal mode observed in this phase, we add a phenomenological gap term, ${\mathrm{\Delta }}_{\mathrm{M}}^2$, to Eq. (5), resulting in a dispersion, $E_{{\mathrm{mode}}}( {{\bf{q}}_{HH}} )^2$ = ${\mathrm{\Delta }}_{\mathrm{M}}^2( {1 - \frac{1}{{3{\cal J}_{{\mathrm{tot}}}^ \bot }}\mathop {\sum}\nolimits_{n = 1}^5 {\cal J}_n^ \bot {\mathrm{cos}}( {2\pi n{\bf{q}}_{HH}} )} )$, and we normalize the couplings to the total interchain exchange, ${\cal J}_{{\mathrm{tot}}}^ \bot = \mathop {\sum}\nolimits_{n = 1}^5 {\kern 1pt} {\cal J}_n^ \bot$. We fit this expression to the measured modes by varying the relative couplings ${\cal J}_n^ \bot {\mathrm{/}}{\cal J}_{{\mathrm{tot}}}^ \bot$, resulting in excellent agreement at all fields (Fig. 3b–d). Increasing the field from 1.0 to 1.5 T dramatically changes the relative interchain coupling strengths. The nearest neighbor coupling is reduced by approximately a factor of 11 when the field is increased from 1.0 to 1.5 T, while the next nearest neighbor coupling is reduced by a factor of 2 and changes sign from ferromagnetic to antiferromagnetic. Smaller changes are found in the higher order terms. These changes are consistent with the observation from the magnetic diffraction (Fig. 4) of a tendency towards a weaker, frustrated longitudinal antiferromagnetic order as the applied magnetic field progressively polarizes the moments (See Supplementary Note 7).

## Discussion

It is difficult to visualize the nature of the interchain mode itself, as it is purely quantum mechanical in its origin, with no simple classical analog. In a conventional 3D ordered magnet, these interchain excitations would be transverse spin waves–pseudo-Goldstone modes of an antiferrromagneic order parameter, acquiring a small gap (mass) in the presence of spin anisotropy. The longitudinal polarization reveals that the new excitations observed here in Yb_2_Pt_2_Pb, which are separated from *E* = 0 with a field-dependent gap *δ* < 0.12 meV, are in fact far more exotic. They represent amplitude excitations of the AFM order parameter, i.e., the staggered magnetization^16,17^, analogous to the amplitude modes of the superconducting order parameter found in NbSe_2_^35,36^ and the Higgs boson^18^ itself. There is a large literature on the subject in the context of the theory of quantum magnets (e.g., refs. ^33,34,37,38^), but so far there have been few experiments among weakly coupled chain systems that probe this mode and its dispersion across different regimes of interchain coupling, or its decay into transverse magnons in detail^39^. This damping causes the longitudinal mode to appear as a resonance in the longitudinally polarized continuum rather than the dispersing quasiparticle like excitation observed here in Yb_2_Pt_2_Pb^6,40^, which often obscures the physics entirely and has even led some to question the assumptions behind the theory^41^. Recently, some evidence for a sharp longitudinal mode coexisting with the transverse spin waves has been obtained via polarized neutron scattering measurements in a more strongly coupled 2D ladder system^42^, while other materials with similar *XXZ* anisotropy to Yb_2_Pt_2_Pb tend to confine all spinons into many modes^11–15^. Here we overcome the limitations of such experiments thanks to the tuning parameters of Yb_2_Pt_2_Pb, which uniquely single out the longitudinal channel and allow us to clearly identify the dispersing amplitude mode and the deconfined 1D excitations at higher energies.

The physics of Yb_2_Pt_2_Pb that suppresses transverse spin waves also protects these longitudinal excitations and allows detailed observation of this mode dispersion and its dependence on an applied magnetic field, which tunes the ordered state across different phases. Accompanying transverse excitations—the spin waves—must exist, but are not observed (Fig. 3f). They are suppressed by a factor (*g*_||_/*g*_⊥_)^2^ ≳ 100 and can not be measured in this scattering geometry, where even the application of a substantial magnetic field leaves behind the longitudinal continuum from magnetic moments perpendicular to the field. While further experiments are needed in alternate scattering geometries to clearly observe the accompanying transverse excitations, the present results quantify in unique detail an unusual dispersing longitudinal mode, a Higgs-like excitation of a effective spin-1/2 order parameter across different ordered phases induced by magnetic fields in Yb_2_Pt_2_Pb.

## Methods

### Neutron scattering

The Yb_2_Pt_2_Pb sample used in these measurements was the same sample used in ref. ^26^. The sample consists of approximately 400 co-aligned Yb_2_Pt_2_Pb single crystals (total mass ≈6 g) mounted to aluminum plates. For all measurements the sample was oriented with the (1${\bar{\mathrm 1}}$0) crystal direction vertical leaving the (110) and (001) directions in the horizontal scattering plane, making the scattering plane (*H*, *H*, *L*) in reciprocal space. All momenta are given in reciprocal lattice units (rlu), with 1 rlu given by 2*π*/*a* = 2*π*/7.76 Å = 0.810 Å^−1^ along the *H* direction and 2*π*/*c* = 2*π*/7.02 Å = 0.895 Å^−1^ along *L*. The crystallographic unit cell is twice the Yb-Yb near neighbor spacing along the *c*-axis—the relevant spacing for spinons. Therefore, the Brillouin zone for spinons is indexed from 0 to 2 rlu along (0, 0, *L*), rather than the typical 0 to 1 rlu. Notationally, **q**_*HH*_ is parallel to the (*H*, *H*, 0) direction, with scattering primarily coming from (*H*, *H*, 1) while **q**_*L*_ is along the (0, 0, *L*) direction.

The inelastic neutron scattering measurements on Yb_2_Pt_2_Pb making up the bulk of the data in this paper were made on the OSIRIS spectrometer at the ISIS neutron source at Rutherford Appleton Laboratory in Didcot, Oxfordshire, UK^43^. The sample was mounted in a dilution refrigerator inside of a 7 T superconducting magnet with the field in the vertical direction parallel to the (1${\bar{\mathrm 1}}$0) crystal direction. OSIRIS is an indirect geometry, time-of-flight neutron spectrometer. The incident neutron beam is a white beam of cold neutrons. The PG002 analyzer was used with a final neutron energy *E*_*f*_ = 1.84 meV (*λ* = 6.67 Å). The energy resolution was ≈0.03 meV at *E* = 0 meV. Due to the magnet construction, only scattering into a range of angles 45° < 2*θ* < 135° is permitted. We therefore discard the 14 detector channels at the lowest scattering angles and the 3 channels at the highest scattering angles, determined by measurements of a vanadium standard in the magnet. All data were corrected for the Yb^3+^ form factor^44^.

The measurements on OSIRIS were made at *T* = 0.140 K. A field greater than 2.3 T along the (1${\bar{\mathrm 1}}$0) crystal direction polarizes all of the magnetic moments parallel to that direction, while leaving the orthogonal moments along the (110) direction in the horizontal plane unaffected due to the ground state doublet of the Yb ions^26^. Measurements made at *μ*_0_*H* = 4 T can therefore be used as a background for measurements made at *μ*_0_*H* < 2.3 T, isolating only the lower field scattering contribution from magnetic moments oriented parallel to the field. All neutron scattering results from OSIRIS have a measurement made at *T* = 0.140 K and *μ*_0_*H* = 4 T subtracted in this fashion. For the nominal zero field measurements, a small bias field of 0.025 T was used to suppress superconductivity in the aluminum sample holder.

In general, inelastic neutron scattering probes the dynamical spin correlation function^27^. Because of the crystal field ground state doublet of the Yb ions in Yb_2_Pt_2_Pb we are sensitive only to the longitudinal component of this function (see Supplementary Note 5).

Because our detector coverage does not include an entire Brillouin zone, (0 < **q**_*L*_ < 2 rlu, 0 < **q**_*HH*_ < 1 rlu) we integrate all data in the scattering plane in the reciprocal space direction orthogonal to the direction being considered. The **q**_*L*_ dependencies show in Figs. 1b and 2d–f (And Supplementary Fig. 1) integrate the entire measured range of **q**_*HH*_. The same **q**_*HH*_ integration was used to demonstrate the amount of low energy spectral weight in the bound state (main text Fig. 3e inset). Similarly, all **q**_*L*_ were integrated to examine the **q**_*HH*_ dependence in Figs. 1c and 3b–d (and Supplementary Figs. 4 and 5) and in the cuts used to extract the interchain mode dispersion in Fig. 3e. Although our data from OSIRIS do not cover an entire Brillouin zone, the symmetry of our measurements about both **q**_*L*_ = 1 rlu and **q**_*HH*_ = 0 rlu confirms that our coverage is sufficient.

Inelastic scattering measurements of the polarization factor (main text, Fig. 3f) and diffraction measurements (main text, Fig. 4a–g) were performed on the Disk Chopper Spectrometer (DCS) at the Center for Neutron Research at the National Institute for Standards and Technology in Gaithersburg, MD, USA^45^. For these measurements, the same sample was mounted in the same scattering geometry as the OSIRIS experiments, inside of a dilution refrigerator inside of a 10 T vertical superconducting magnet. DCS is a direct geometry spectrometer and *E*_*i*_ = 3.27 meV (*λ* = 5.00 Å) was used. The energy resolution was ≈0.1 meV. The temperature of these measurements was *T* = 0.07 K. Measurements on DCS have a similarly measured background subtracted and the scattering was corrected for the Yb^3+^ form factor^44^ and neutron absorption using the DAVE software package^46^ in the same manner described in the Supplementary Materials for^26^. The measurement at *μ*_0_*H* = 4 T of the polarization factor (main text Fig. 3f) is simply the *μ*_0_*H* = 4 T measurement on its own, with no background subtracted. For the nominal zero field measurements, a small bias field of 0.025 T was used to suppress superconductivity in the aluminum sample holder.

### Specific heat and magnetization

Specific heat measurements used in the phase diagram shown in Fig. 3a (and Supplementary Figs. 2 and 3) were made using a Physical Property Measurement System (PPMS) made by Quantum Designs on a single crystal of Yb_2_Pt_2_Pb (note: the identification of the equipment used in the various measurements is not intended to imply recommendation or endorsement by the National Institute of Standards and Technology, nor is it intended to imply that this equipment is necessarily the best available for the purpose.) Measurements for *T* < 1.8 K utilized a PPMS dilution refrigerator insert. The (110) direction was oriented vertically, parallel to the magnetic field. Magnetization measurements for *T* < 1.8 K in Figs. 2a and 4e (and Supplementary Fig. 6) were made using a PPMS Hall sensor magnetometer in a dilution refrigerator insert at *T* = 0.150 K. Magnetization measurements for *T* > 1.8 K in Fig. 3a were made using a Quantum Designs Magnetic Property Measurement System SQUID magnetometer. The magnetic field for both sets of measurements was along the (110) direction.

### Obtaining the position of the interchain mode

The interchain mode dispersions shown in Fig. 3b–d (and Supplementary Fig. 5) were extracted by integrating a window of **q**_*HH*_ = 0.1 rlu and fitting the resulting cuts of the scattering function as a function of energy *S*(*E*), examples of which are shown in Fig. 3e of the main text. The fitting function represents the mode as a damped harmonic oscillator – the product of the Bose population factor with the difference of two Lorentzians, centered at positive and negative energies *E*_mode_, with a width Γ and amplitude *A*, Eq. (6).

6 $$I\left( E \right) = \frac{A}{{1 - e^{ - E/k_{\mathrm{B}}T}}}\left( {\frac{\Gamma }{{\left( {E - E_{{\mathrm{mode}}}} \right)^2 + {\mathrm{\Gamma }}^2}} - \frac{\Gamma }{{\left( {E + E_{{\mathrm{mode}}}} \right)^2 + {\mathrm{\Gamma }}^2}}} \right)$$

This function was added to the product of another Lorentzian and step function each centered at energy *E* > *E*_mode_ representing the continuum. The fit itself was performed to these two functions convolved with a gaussian representing the instrumental resolution.

### Time-dependent density matrix renormalization group calculations

The longitudinal component of the dynamical spin structure factor *S*(**q**, *ω*) was obtained by means of the time-dependent density matrix renormalization group (tDMRG) method^28,47–49^. The approach has been extensively described in the literature and essentially consists of calculating the time-dependent correlation function:

7 $$S\left( {x - x_0,t} \right) = \left\langle \psi \right|e^{i\hat Ht}\hat S^z(x)e^{ - i\hat Ht}\hat S^z\left( {x_0} \right)\left| \psi \right\rangle .$$

The operator $\hat S^z(x_0 = L{\mathrm{/}}2)$ is applied at the center of the chain and the resulting state is evolved in real-time. At every step, the overlap with the state $\hat S^z(x)e^{ - i\hat Ht}\left| \psi \right\rangle$ is measured and the correlations function in real time and space is recorded. The results are Fourier transformed to frequency and momentum using a properly chosen Hann window that determines the resolution of the final result. In our case, in order to compare to experiments we have used a window of half-width Δ*t* = 7 in units of 1/*J*. Only two things differ from conventional calculations: since the parameters considered fall into the Ising phase of the model, which tends to break translational symmetry at finite magnetization, the simulations were carried out using periodic boundary conditions and the time-targeting method with a Krylov expansion of the evolution operator^50^. In addition, we plot the results for the operator $\left( {\hat S^z(x) - m{\mathrm{/2}}} \right)$ (instead of $\hat S^z(x)$), where *m* is the magnetization value. The offset allows us to resolve the density fluctuations and eliminates large contributions at low frequencies and momenta^51^. Surprisingly, due to the low entanglement in the Ising phase, truncation errors smaller than 10^−5^ are easily achievable using 300 states, even on a chain of length *L* = 96 with periodic boundary conditions.

The color scale for the calculations displayed in Figs. 1d and 2g–i was determined by normalizing the integral of the *E* > 0 portion of the calculation to the measured integral of the inelastic intensity at the the same field.

## Supplementary information

Supplementary Information

## Data Availability

The data that support the findings of this study are available from the corresponding authors upon reasonable request. Original time-of-flight data is available for the experiments at the ISIS neutron source at 10.5286/ISIS.E.42580328.
