# Supplementary material for: Spinon confinement and a sharp longitudinal mode in Yb2Pt2Pb in magnetic fields
Source: Nat Commun. 2019 Mar 8;10:1123. doi: 10.1038/s41467-019-08715-y (PMC6408591; doi:10.1038/s41467-019-08715-y)
Supplement: Supplementary file 1 — Supplementary Information [file 41467_2019_8715_MOESM1_ESM.pdf]

Supplementary Information

Spinon Confinement and a Sharp Longitudinal Mode in Yb_2Pt_2Pb in Magnetic Fields

W. J. Gannon *et al.*

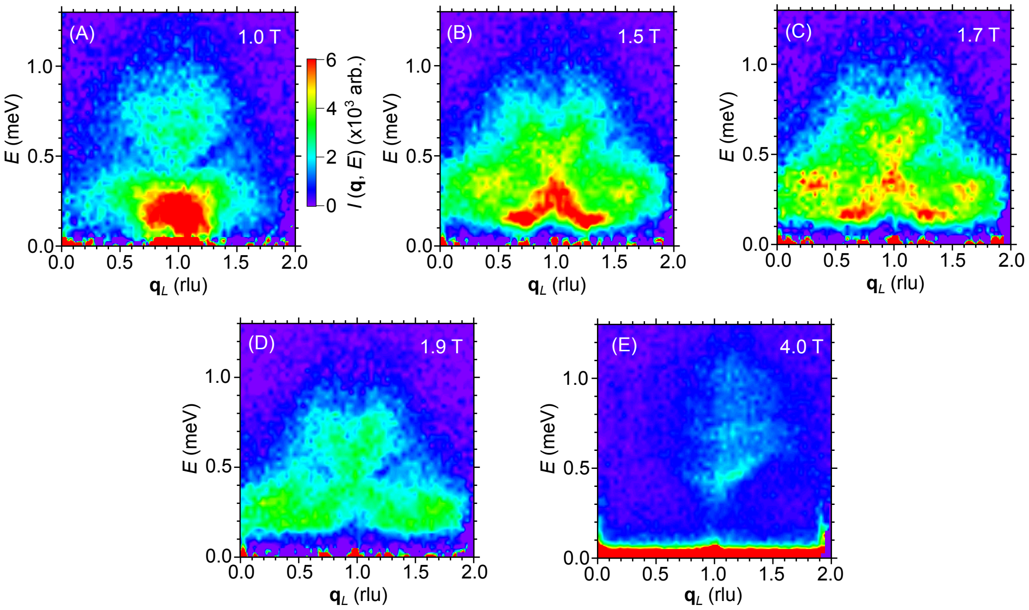

FIG. 1. **Spinons in Yb_2Pt_2Pb in Magnetic Field**. The spectra for momentum along the chain direction $\mathbf{q}_L$ for fields of (A) $\mu_0 H = 1.0$ T, (B) 1.5 T, (C) 1.7 T, (D) 1.9 T, and (E) 4.0 T. Color scale is the same for all panels as part (A). Data in panels A-D are the same as Fig. 2 of the main text, but without the continuum boundaries.

SUPPLEMENTARY NOTE 1

The observation of a continuum of scattering for all fields $\mu_0 H < 2.3$ T necessarily indicates the presence of multi spinon states. The energy of any two spinon state is given by the sum of the two spinon energies while the wavevector of the two spinon state is given by the vector sum of the momenta of each. The energy and momentum of the two spinon state must conserved with respect to the change in energy and momentum between the incident neutron's initial and final states. The possible energy and momentum of each spinon is given by the single spinon dispersions

$$E_{\mathrm{p,h}}(\mathbf{q}) = \pm \left(I^2 \sin^2(\pi \mathbf{q}_L) + \Delta_{\mathrm{S}}^2 \cos^2(\pi \mathbf{q}_L)\right)^{1/2} \tag{1}$$

where $E_{\mathrm{p,h}}$ is the energy for a "particle" and "hole," *i.e.* a spin-up spinon and a spin-down spinon. As discussed in the main text, $I = 0.485$ meV and $\Delta_{\mathrm{S}} = 0.095$ meV for Yb_2Pt_2Pb.

The envelope of the continuum is determined by the extremal two spinon states. In zero field, there are three extremal cases. In one case, if the hole momentum $\mathbf{q}_{\mathrm{h}} = 0$, all of the momentum $\mathbf{q}_{\mathrm{2spin}} = \mathbf{q}_{\mathrm{p}} + \mathbf{q}_{\mathrm{h}}$ of the two spinon state will be carried by the particle, $\mathbf{q}_{\mathrm{2spin}} = \mathbf{q}_{\mathrm{p}}$. The energy of the two spinon state E_{2spin} will given by $E_{\mathrm{2spin}} = E_{\mathrm{h}}(0) + E_{\mathrm{p}}(\mathbf{q}_{\mathrm{2spin}})$. This is degenerate with the case where $\mathbf{q}_{\mathrm{2spin}} = \mathbf{q}_{\mathrm{h}}$ and $\mathbf{q}_{\mathrm{p}} = 0$. The third case has the particle and hole sharing the momentum equally, $\mathbf{q}_{\mathrm{p}} = \mathbf{q}_{\mathrm{h}} = \mathbf{q}_{\mathrm{2spin}}/2$, so that the two spinon dispersion is given by $E_{\mathrm{2spin}} = E_{\mathrm{p}}(\mathbf{q}_{\mathrm{2spin}}/2) + E_{\mathrm{h}}(\mathbf{q}_{\mathrm{2spin}}/2)$. All other two spinon states have energies that fall between these possibilities. They are sketched in Fig.1(B,D) of the main text.

Application of a field along the z direction large enough for the Zeeman energy $g\mu_B H S^z$ to close the gap Δ_{S} in the single spinon dispersion changes the extremal combinations of two spinons. There are far more possible two spinon states, since there are now more routes to creating particle-hole paris as the hole (or particle, depending on the sign convention) dispersion is emptied with increasing field. The boundaries of the two spinon continuum that are sketched in Fig. 2(D-I) of the main text are arrived at by fixing the particles (holes) at momentum $\mathbf{k}_{\mathrm{F}}$ and $1-\mathbf{k}_{\mathrm{F}}$ and creating excitations with holes (praticles) at $\mathbf{k}_{\mathrm{F}}$ and $1-\mathbf{k}_{\mathrm{F}}$, fixing the particle to have 0 momentum, fixing the particle to have momentum $\mathbf{q}_{\mathrm{p}} = 1$ and allowing the particle and hole to share the momentum equally.

SUPPLEMENTARY NOTE 2

The spectra for momentum along the chain direction $\mathbf{q}_L$ in a magnetic field (main text, Fig. 2(D-F)) are presented in Fig. 1(A-D) without the continuum boundaries. Data at $\mu_0 H = 1.9$ T are also shown. The spectrum at 1.9 T is consistent with 1.5 and 1.7 T – all are in the same antiferromagnetic phase. The 4.0 T data in Fig. 1(E) are the same

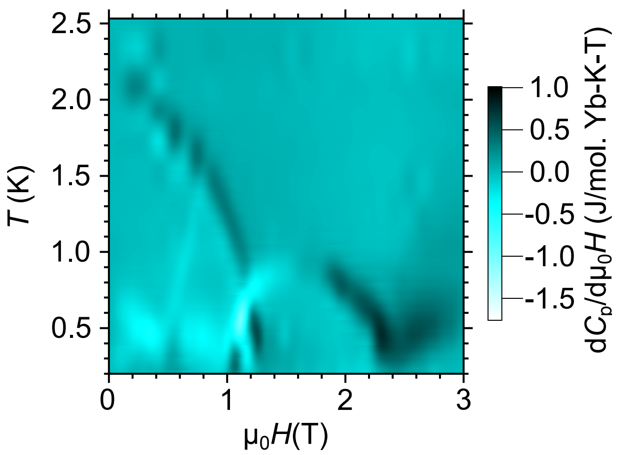

FIG. 2. **Antiferromagnetic Phase Diagram of Yb_2Pt_2Pb**. The antiferromagnetic phase diagram of Yb_2Pt_2Pb from measurements of the field derivative of the specific heat, an example of which is shown in Fig. 3.

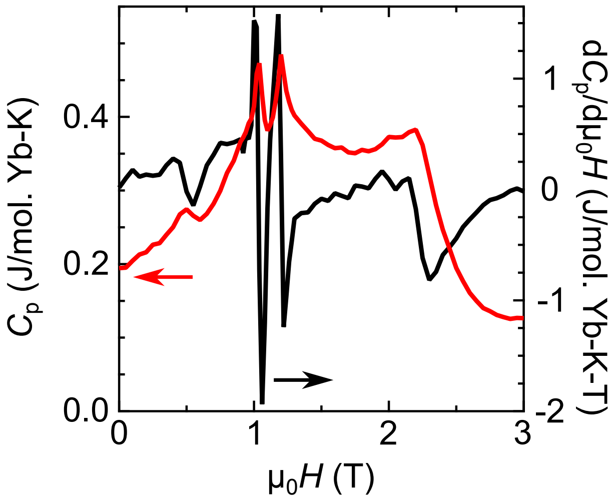

FIG. 3. **Specific Heat of Yb_2Pt_2Pb**. The magnetic field dependence of the specific heat C_p of Yb_2Pt_2Pb measured at $T = 0.35$ K (red, left axis). Also shown are the field derivative of the specific heat $\mathrm{d}C_\mathrm{p}/\mathrm{d}\mu_0 H$ (black, right axis) at the same temperature.

as in zero field, but with a $\mathbf{q}_L$ dependence that reflects the polarization factor of the Ising magnetic moments in the scattering plane, perpendicular to the applied magnetic field.

SUPPLEMENTARY NOTE 3

The color plot used in the antiferromagnetic phase diagram shown in Fig. 3 of the main text was obtained from the specific heat. We present the same color plot here in Fig. 2 without the phase lines determined from magnetization. Measurements of the specific heat C_p were made at many fixed temperatures as a function of a magnetic field parallel to the crystal (110) direction. An example of such a measurement at $T = 0.35$ K is shown in Fig. 3. To enhance contrast for the color plot, the quantity plotted in the phase digram is the field derivative of the specific heat, $\mathrm{d}C_\mathrm{p}/\mathrm{d}\mu_0 H$. An example of the derivative at $T = 0.35$ K is shown in Fig. 3. Many such measurements are combined to make Fig. 2, which compares well with the phase diagrams determined previously. [1–3]

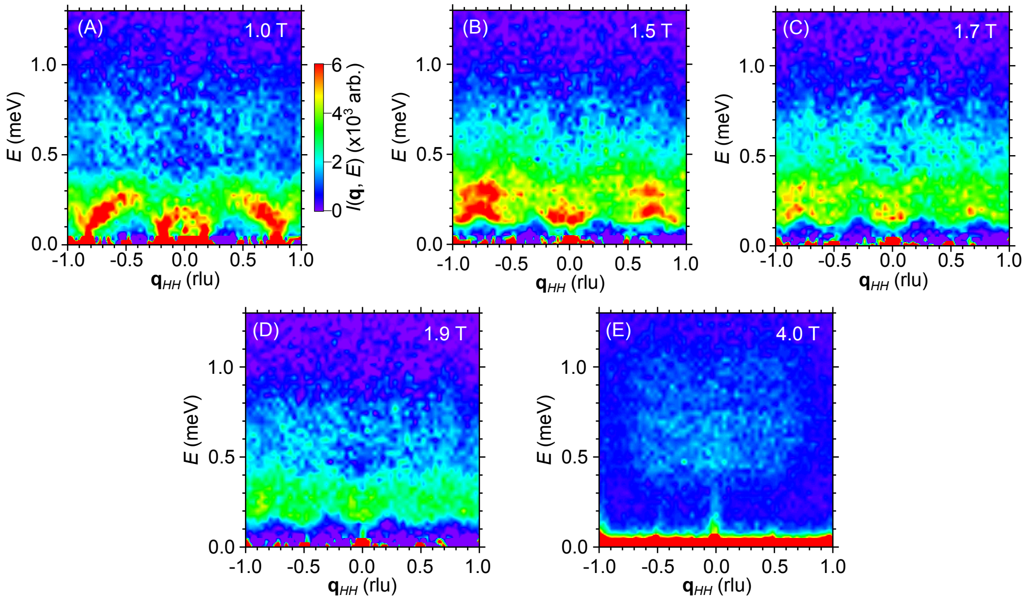

FIG. 4. **Evolution of Interchain mode in Yb_2Pt_2Pb**. The spectra for momentum perpendicular to the chain direction $\mathbf{q}_{HH}$ for fields of (A) $\mu_0 H = 1.0$ T, (B) 1.5 T, (C) 1.7 T, (D) 1.9 T, and (E) 4.0 T. Color scale is the same for all panels as part (A). Data in panels A-D are the same as Fig. 3 of the main text, but the mode position and dispersion fits are not shown.

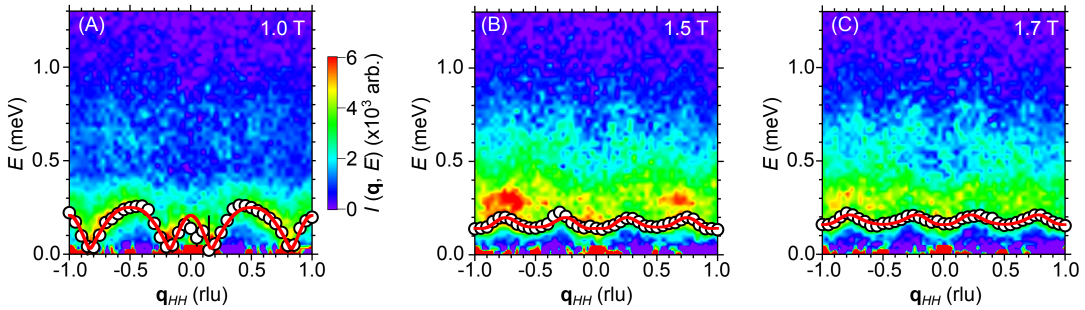

FIG. 5. **Evolution of Interchain mode in Yb_2Pt_2Pb**. The spectra for momentum perpendicular to the chain direction $\mathbf{q}_{HH}$ for fields of (A) $\mu_0 H = 1.0$ T, (B) 1.5 T, and (C) 1.7 T. Color scale is the same for all panels as part (A). Data are the same as Fig. 3 of the main text, but the mode position and dispersion fits are shown from $-1 < \mathbf{q}_{HH} < 1$ rlu. Error bars represent one standard deviation.

SUPPLEMENTARY NOTE 4

The spectra for momentum along $\mathbf{q}_{HH}$, perpendicular to the chain direction (main text, Fig. 3(B-D)) are shown in Fig. 4(A-D) without the mode position or dispersion fits. Data at $\mu_0 H = 1.9$ T are also shown. The spectrum at 1.9 T is consistent with 1.5 and 1.7 T and is not shown in the main text. The 4.0 T data in Fig. 1(E) are the same as in zero field, but with a $\mathbf{q}_{HH}$ dependence that reflects the polarization factor of the Ising magnetic moments in the scattering plane, perpendicular to the applied magnetic field. In Fig. 5, we show the same spectra with the mode position and dispersion fit over the entire measured range of $\mathbf{q}_{HH}$. The dispersion and fits shown in Fig.3(B-D) of the main text are the same, but are only displayed for $\mathbf{q}_{HH} > 0$ in order to show both the mode in the color plot and in the analyzed data simultaneously. The fits themselves were performed over the entire range of $\mathbf{q}_{HH}$.

SUPPLEMENTARY NOTE 5

The scattering geometry of our measurements orients the $(1\bar{1}0)$ crystal direction vertically, parallel to the magnetic field, with the (110) direction in the horizontal scattering plane. Measurements made in a field $\mu_0 H > 2.3$ T

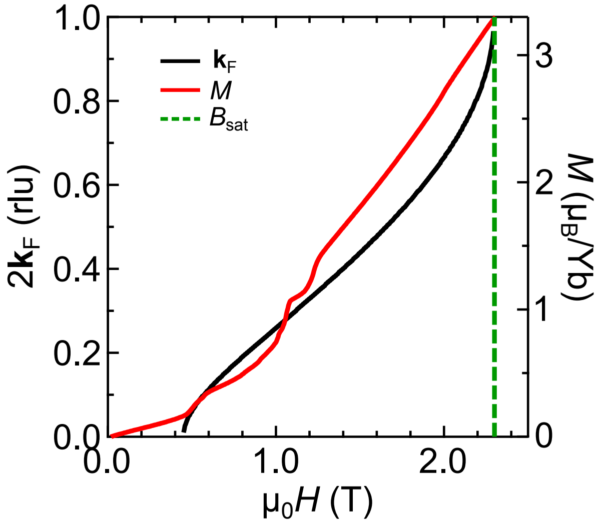

FIG. 6. **Fermi wavevector and Magnetization**. Two times the fermi wavevector $\mathbf{k}_{\mathrm{F}}$ (black, left axis) and the magnetization M (red, right axis) as a function of magnetic field along the (110) direction. Magnetization was measured at $T = 0.150$ K. The saturation field (green dashed line) is at $\mu_0 H_{\mathrm{sat}} = 2.3$ T.

will quench all excitations in the Yb sublattice with moments oriented along $(1\bar{1}0)$, while having no effect on the perpendicular sublattice. Using such a measurement as a background for lower fields isolates the fluctuations parallel to $(1\bar{1}0)$. Plotting the total scattered intensity as a function of $\mathbf{q}_{HH}$ at $\mathbf{q}_L= 1$ (main text, Fig. 3(F)) demonstrates the wavevector dependence of the scattering as $\mathbf{q}$ is rotated from the c-axis, $(\mathbf{q}_{HH} = 0)$ towards the (110) direction $(\mathbf{q}_{HH} \gg \mathbf{q}_L)$. We find a constant scattered intensity, and since neutrons only scatter from components of the local magnetization density perpendicular to $\mathbf{q}$, [4] this demonstrates that the fluctuating magnetic moments always have the same projection on the scattering vector and are thus always perpendicular to $\mathbf{q}$. The same data plotted for a fixed field of 4 T show that the scattering precisely follows the geometric projection of the moments in the scattering plane onto the scattering vector, $I \propto \mathbf{q}_L^2/(2\mathbf{q}_H^2 + \mathbf{q}_L^2)$, providing further confirmation that the fluctuations are purely longitudinal.

SUPPLEMENTARY NOTE 6

The field dependence of the magnetization matches the field dependence of $2\mathbf{k}_{\mathrm{F}}$ in 1D systems [5]. In Fig. 6, we show the correspondence between the magnetization measured at $T = 0.150$ K, below the spinon gap Δ_{S} with $2\mathbf{k}_{\mathrm{F}}$ calculated from where the chemical potential $g\mu_B H S^z$ crosses the single spinon dispersions, given for particles and holes by Eqn. 1 with $I = 0.485$ meV and $\Delta_{\mathrm{S}} = 0.095$ meV. The magnetization has several kinks corresponding to metastability in the underlying incommensurate antiferromagnetic order, demonstrated in Fig.4(E,F) of the main text.

SUPPLEMENTARY NOTE 7

Our description of the longitudinal interchain mode requires fitting the strengths of the relevant interchain couplings, $\mathcal{J}_n^{\perp}$, with $n = 1$ for nearest neighbor coupling, $n = 2$ for next nearest neighbors, *etc* [6]. The fitting function must be normalized to the total interchain exchange couplings $\mathcal{J}_{\mathrm{tot}}^{\perp} = \sum_{n=1}^{5} \mathcal{J}_n^{\perp}$ and include a gap Δ_{M}. We give the dispersion by $E_{\mathrm{mode}}(\mathbf{q}_{HH})^2 = \Delta_{\mathrm{M}}^2 \left(1 - \frac{1}{3\mathcal{J}_{\mathrm{tot}}^{\perp}} \sum_{n=1}^{5} \mathcal{J}_n^{\perp} \cos(2\pi n \mathbf{q}_{HH})\right)$, which includes 5 terms in the sum to account for the periodicity of the underlying antiferromagnetic order. Defining $\mathcal{J}_n^{\mathrm{rel}} = \mathcal{J}_n^{\perp}/\mathcal{J}_{\mathrm{tot}}^{\perp}$, we vary $\mathcal{J}_n^{\mathrm{rel}}$ in our fits. The results of the fit, along with the standard deviations of the fits are given in Table 1.

$\mu_0 H$ (T)	Δ_{M} (meV)	$\mathcal{J}_1^{\mathrm{rel}}$	$\mathcal{J}_2^{\mathrm{rel}}$	$\mathcal{J}_3^{\mathrm{rel}}$	$\mathcal{J}_4^{\mathrm{rel}}$	$\mathcal{J}_5^{\mathrm{rel}}$
1.0	0.174 ± 0.001	1.96 ± 0.09	-1.96 ± 0.09	-0.882 ± 0.07	-0.248 ± 0.06	-0.119 ± 0.06
1.5	0.167 ± 0.0006	0.171 ± 0.03	1.01 ± 0.03	-0.0433 ± 0.03	-0.263 ± 0.03	-0.0258 ± 0.03
1.7	0.182 ± 0.0007	-0.100 ± 0.03	0.826 ± 0.03	0.190 ± 0.03	-0.119 ± 0.03	-0.0734 ± 0.03

TABLE 1. Relative strength of the interchain couplings giving the longitudinal interchain mode dispersion.

[1] Ochiai, A. *et al.* Field-induced partially disordered state in Yb_2Pt_2Pb. *Journal of the Physical Society of Japan* **80**, 123705(1–4) (2011).

[2] Shimura, Y., Sakakibara, T., Iwakawa, K., Sugiyama, K. & Onuki, Y. Low temperature magnetization of Yb_2Pt_2Pb with the shastry-sutherland type lattice and a high-rank multipole interaction. *Journal of the Physical Society of Japan* **81**, 103601(1–4) (2012).

[3] Kim, M. S. & Aronson, M. C. Spin liquids and antiferromagnetic order in the shastry-sutherland-lattice compound Yb_2Pt_2Pb. *Physical Review Letters* **110**, 017201(1–6) (2013).

[4] Squires, G. L. *Introduction to the Theory of Thermal Neutron Scattering* (Cambridge University Press, England, 2012), third edition edn.

[5] Giamarchi, T. *Quantum Physics in One Dimension* (Oxford Science Publications, 2004).

[6] Schulz, H. J. Dynamics of coupled quantum spin chains. *Physical Review Letters* **77**, 2790–2793 (1996).
